# Safety and Efficacy of a Selective Inhibitor of Cyclin-dependent Kinase 9 (KB-0742) in Patients with Recurrent or Metastatic Adenoid Cystic Carcinoma

**DOI:** 10.1158/2767-9764.CRC-25-0015

**Published:** 2025-05-07

**Authors:** Glenn J. Hanna, Gregory M. Cote, Rashmi Chugh, Jacob S. Thomas, Jyoti Malhotra, Richard E. Cutler, Tressa Hood, Luis A. Carvajal, Elizabeth A. Olek, Miguel A. Villalona-Calero

**Affiliations:** 1 Center for Head and Neck Oncology, Dana-Farber Cancer Institute, Boston, Massachusetts.; 2 Mass General Cancer Center, Boston, Massachusetts.; 3 Rogel Cancer Center, University of Michigan, Ann Arbor, Michigan.; 4 Keck School of Medicine of USC, Los Angeles, California.; 5 City of Hope Orange County, Irvine, California.; 6 City of Hope National Medical Center, Duarte, California.; 7 Kronos Bio, Inc., San Mateo, California.; 8 UCI Health Chao Family Comprehensive Cancer Center, Orange, California.

## Abstract

**Purpose:**

Adenoid cystic carcinoma (ACC) is a rare salivary gland malignancy of the head and neck. Recurrent or metastatic ACC has very limited therapeutic options. Cyclin-dependent kinase 9 (CDK9) is a key factor in the oncogenic transcriptional regulatory network, and inhibition of CDK9 may prove beneficial in *MYC*-dependent tumors such as ACC.

**Patients and Methods:**

A first-in-human, phase I, two-part dose-escalation and -expansion clinical trial (NCT04718675) enrolled patients with advanced solid tumors reliant on transcription factor activation to receive KB-0742, an oral selective inhibitor of CDK9. The primary endpoint was to establish safety/tolerability while nominating a recommended phase II dose (RP2D). Secondary endpoints included characterization of pharmacokinetics and assessment of preliminary efficacy.

**Results:**

Among 19 patients with ACC enrolled in dose expansion at the RP2D (60 mg orally 3 days on and 4 days off each week during a 28-day cycle), the regimen was well tolerated with mild gastrointestinal toxicity and fatigue; a single grade 3 treatment-related adverse event was observed (elevated γ-glutamyl transferase). One patient discontinued for gastrointestinal toxicity. Although no responses were observed, nine of 16 (56%) eligible patients had stable disease, with four experiencing >6 months of stability. Six-month progression-free survival was 37% (95% confidence interval, 14.2–59.8). Most patients had the more indolent type II ACC phenotype and 10 (53%) had *MYB* alterations.

**Conclusions:**

This dose-expansion cohort exploring the novel CDK9 inhibitor KB-0742 in patients with advanced ACC established favorable tolerability at the RP2D. Disease stabilization was observed in some patients despite a limited efficacy signal.

**Significance:**

A first-in-human, phase I trial explored the safety and preliminary efficacy of the CDK9 inhibitor KB-0742 in patients with advanced, transcription factor–dependent solid tumors including ACC. KB-0742 was well tolerated with evidence of disease stabilization observed among some patients with ACC, but overall therapeutic efficacy was limited.

## Introduction

Adenoid cystic carcinoma (ACC) is a relatively uncommon cancer, accounting for 1% of all head and neck malignancies, but represents the most common malignant tumor arising in the minor salivary glands (1). Although rare, the clinical course of ACC is typically characterized by perineural invasion and hematogenous spread despite aggressive attempts at locoregional control with surgery and radiation (2). Patients often present with late distant recurrences in the lung and elsewhere, representing advanced incurable disease which can have a relentless but often indolent growth pattern.

Treatment options for recurrent or metastatic ACC are limited by modest antitumor activity and tolerability concerns despite years of investigation aimed at identifying preferred therapies (3). Studies have shown some signal of activity with platinum-based cytotoxic chemotherapy regimens and VEGFR-targeting tyrosine kinase inhibitors (TKI; refs. 4–7), but toxicity issues remain a concern. Immunotherapy can be an option but has been associated with minimal clinical benefit in ACC (8, 9).

Broader tumor genomic profiling efforts have nominated key molecular targets of interest in ACC including Notch, chromatin remodeling genes, and *TERT* promoter mutations (10). More recent molecular profiling efforts have identified two important ACC subtypes: a more aggressive ACC-I defined by activating Notch pathway alterations and *MYC* amplification, and the more common ACC-II which demonstrates TP63 overexpression yielding a more indolent phenotype (11, 12). Regardless of ACC subtype, the most prevalent genomic alteration is a recurrent chromosomal translocation involving the *MYB* oncogene and NFIB transcription factor (TF). *MYB–NFIB* fusion triggers enhanced TF activity to control cell-cycle progression and antiapoptotic signaling, leading to cancer cell survival (13). With greater awareness of these molecular observations in ACC, these targets have been the focus of recent clinical trials for patients with recurrent or metastatic disease.

Cyclin-dependent kinase 9 (CDK9) is a crucial serine/threonine kinase that is primarily involved in regulating transcription elongation during gene expression (14). It is part of the CDK family which consists of key regulators of the cell cycle, but CDK9 is also involved in transcription of oncogenes. CDK9 is required for the transcription of *MYC*, a key oncogene that regulates cell growth in many cancers including ACC (15). KB-0742 is an orally available, selective inhibitor of CDK9 that competes with ATP binding at its activation site, resulting in loss of CDK9 kinase activity and disruption of downstream transcription. Many first-generation CDK9 inhibitors inhibit cell cycle CDKs within 10× of their CDK9 IC_50_, leading to off-target toxicity, whereas KB-0742 is at least 66× selective for CDK9 over cell cycle CDKs.

Here, we present dose-expansion cohort results of a first-in-human clinical study investigating KB-0742. Although the trial included many *MYC*-dependent tumor types in which CDK9 acts as a central focus in the oncogenic transcriptional regulatory network, this report focuses on the treated ACC subgroup.

## Materials and Methods

### Study oversight

The study protocol (see Supplementary Appendix S1) was approved by the institutional review boards at all participating centers, and the trial is registered with ClinicalTrials.gov (NCT04718675). The trial was conducted in accordance with the principles of the Declaration of Helsinki and the International Conference on Harmonization Good Clinical Practice guidelines. All patients provided written informed consent. Study funding and KB-0742 were provided by Kronos Bio.

### Study design and treatment

This multicenter, non-randomized, first-in-human, open-label, sequential cohort, phase I dose-escalation and -expansion clinical trial enrolled patients at 35 sites across the United States. Following informed consent, patients in part 1 received sequential escalating doses (minimum three patients per dose level with staggered enrollment) of KB-0742 starting at 10 mg orally for 3 consecutive days followed by a 4-day treatment-free interval each week in 28-day cycles. Tolerance permitting, expanded dose regimens were planned with parallel dose-escalation cohorts for separate dosing schedules at equivalent total weekly dosing. Tumor types of interest in part 1 included small cell lung cancer, epithelial ovarian cancer, triple-negative breast cancer, non–small cell lung cancer, epithelial solid tumors with evidence of *MYC* copy-number gain, sarcoma subtypes associated with TF fusion, chordoma, NUT carcinoma, and ACC. Dose escalation proceeded following a modified continuous reassessment method using a Bayesian approach to track the dose-limiting toxicity (DLT) relationship, so as to not exceed a DLT probability of 20% (MTD), and later was replaced with a 3 + 3 design. Backfill was permitted up to 20 participants into a previously cleared dose. A chartered Dose Escalation Committee, comprised of all investigators who enrolled patients, served as safety review for both dose escalation and cumulative safety data review. The totality of pharmacokinetic (PK), pharmacodynamic, and safety data determined the recommended phase II dose (RP2D) in part 2.

Part 2 or dose expansion was planned worldwide. Enrollment with the RP2D dose was planned in cohort A with advanced non–small cell lung cancer, triple-negative breast cancer, and ovarian cancer enrolling up to 40 participants in each subgroup. Cohort B included up to 20 participants with advanced small cell lung cancer and 60 participants collectively with sarcomas with TF fusions, chordoma, NUT carcinoma, and ACC. All patients were treated until documentation of progressive disease (PD), unacceptable toxicity, or study withdrawal.

### Study participants

Patients 18 years (12 years in the United States) or older were eligible if they had relapsed or refractory solid tumors (outlined above) with at least one site of measurable disease by RECIST v1.1, an Eastern Cooperative Oncology Group performance status of 0 to 1, adequate organ and marrow function, and preserved cardiac function. Participants in part 1 had to have one biopsy-accessible site and consent to pre- and on-treatment paired biopsies. Any number of lines of prior systemic therapy for advanced disease were permitted. Disease progression prior to study enrollment was not required although patients had to have failed their most recent line of therapy or not responded. Patients with stable or treated central nervous system disease were eligible. Full eligibility criteria are detailed in the Supplementary Appendix S1.

### Endpoints

The primary endpoint in both part 1 and 2 was to establish safety and tolerability while nominating an RP2D for KB-0742. Secondary endpoints included characterization of the PK of KB-0742 and assessment of preliminary efficacy. Exploratory endpoints were to assess the extent of target engagement at the RP2D, measure baseline *MYC* overexpression, and assess tumor-related molecular profiling data.

### Assessments

Routine laboratory assessments and electrocardiography were performed with each treatment cycle. Adverse events (AE) to assess the safety and side effect profile of treatment were recorded using Common Terminology Criteria for Adverse Events version 5.0. In part 1, a DLT was defined as any grade 4 non-hematologic event, any grade 3 non-hematologic event [except gastrointestinal (GI) toxicity resolving within 3 days and grade 3 rash or fatigue resolving to grade 2 within 4 days with optimized medical management], or hematologic events including grade 4 neutropenia lasting >5 days, febrile neutropenia, and grade 3 thrombocytopenia with significant bleeding – any within the first treatment cycle. AEs were captured to resolution after discontinuation of study drug.

Patients underwent imaging assessments (contrast-enhanced CT of the chest, abdomen, pelvis, and other anatomic locations as appropriate) at baseline and every 8 weeks or two cycles while on treatment. Following treatment discontinuation, patients completed an end-of-study visit and were followed for resolution of toxicity and to document survival. Archival formalin-fixed, paraffin-embedded tissue blocks or a minimum of 20 unstained slides were collected centrally if paired on-treatment biopsies were not feasible to permit molecular profiling. Tumor molecular profiling data were collected before treatment and obtained from any commercial or in-house next-generation sequencing platform (utilized to characterize cases as ACC-I vs. ACC-II based on the presence of a Notch pathway alteration in the appropriate clinical context).

### Statistical design

No specific hypothesis testing was planned in part 1, and descriptive statistics were used to analyze data. The study employed a modified continuous reassessment method design to estimate MTD with Bayesian methodology by continuously assessing DLTs as dose cohort data became available and later changed to a conventional 3 + 3 design when exploring various treatment schedules. This continued until 80 participants had been treated or the MTD had been identified. The safety population included all patients who received ≥1 dose of KB-0742 and had at least one on-treatment safety-related observation. PK parameters were summarized using descriptive statistics.

The efficacy-evaluable population included participants with a baseline assessment of tumor burden and at least one on-study response assessment. Best overall response was summarized by frequency distribution and percentages. The 95% confidence intervals (CI) for estimates of the proportion of subjects in response were constructed with exact methods for the binomial distribution. Progression-free survival (PFS) was defined as the time from cycle 1 day 1 until progression or death from any cause or censored at the last follow-up. The duration of response or stable disease (SD) was defined as the time from the first qualifying event until progression or death. The method of Kaplan and Meier was used to estimate the medians for PFS and duration of response or stability at specified landmark timepoints. Two-sided *P* values are reported. Data as of December 2024 were analyzed using Stata 18 (StataCorp LLC) and GraphPad Prism v10.

### Data availability

Requests for data are limited to those datasets described in this article and not publicly available because of patient privacy concerns. The data generated from this clinical trial are available upon reasonable request from the corresponding author up to 6 years following publication. Tumor genomic sequencing testing relied on commercial assay reporting. Requests for access to raw tumor sequencing data can be submitted directly to commercial entities (Caris Life Sciences, NeoGenomics, or Foundation Medicine).

## Results

### Administrative summary

Between October 2022 and April 2024, 112 patients enrolled to the clinical trial with 42 in dose escalation across five dose cohorts (10, 20, 40, 60, and 80 mg) and 70 in expansion (at 60 mg dosing schedules). Based on the PK in escalation, the study evaluated the antitumor activity of KB-0742 at a dose of 60 mg 3 days on, 4 days off while continuing to dose escalate in parallel to define the MTD which was later defined at 80 mg 4 days on, 3 days off based on the PK.

In total, 19 patients with ACC enrolled to the study. All patients with ACC began protocol treatment and were included in the safety set, whereas three patients were unevaluable and excluded from efficacy analyses for discontinuing treatment before first restaging (two withdrew from study participation and one was lost to follow-up). Eighteen of the 19 (95%) patients with ACC received the 60 mg dose (3 days on, 4 days off) in expansion (part 2, cohort B), and one (5%) received the 80 mg dose in escalation (part 1). In late 2024, the pharmaceutical sponsor deprioritized KB-0742 and terminated the study early. The totality of the part 1 data which included PK and pharmacodynamic studies was limited for inclusion in the present report (16).

### Study participants

The median age was 66 years (range, 50–77) with most identifying as female at birth (10; 53%; Table 1; Supplementary Table S1). Most had a primary tumor that arose in the major salivary glands (14; 74%), and the median time since initial diagnosis to starting study treatment was 3 years (range, <1–12). Nearly all patients had at least one prior therapy for recurrent or metastatic ACC (15, 79%), with three (16%) having had three or more lines. Of the 15 (79%) patients who had prior systemic therapies, nine experienced PD on their immediate prior treatment before enrolling on study. Fifteen (79%) patients had molecular profiling and clinical features suggesting ACC-II disease, whereas four (21%) had the more aggressive ACC-I subtype. Nearly half (10, 53%) of patients had tumors harboring a *MYB–NFIB* fusion.

**Table 1:** Baseline patient characteristics

Characteristic	Total population (%)^a^*N* = 19
Age, years (median)	66 (50–77)
Gender	
Male	9 (47)
Female	10 (53)
Race^b^	
White/Caucasian	19 (100)
Black or African American	0
Asian	0
Ethnicity^b^	
Non-Hispanic	18 (95)
Hispanic	1 (5)
ECOG performance status	
0	9 (47)
1	10 (53)
Primary site of disease	
Major salivary gland (parotid, submandibular, and sublingual)	14 (74)
Minor salivary gland	4 (21)
Glands outside the head and neck	1 (5)
Molecular classification	
ACC type I	4 (21)
ACC type II	15 (79)
*MYB–NFIB* fusion^c^	10 (53)
Years since initial diagnosis (median)	3 (<1–12)
Lines of prior systemic therapy for recurrent or metastatic disease	
0	4 (21)
1	9 (47)
2	3 (16)
3 or more	3 (16)

Abbreviation: ECOG, Eastern Cooperative Oncology Group.

a Values are numbers and percentages, except age and years since initial diagnosis showing range in parentheses.

b As classified by the participant.

c Not mutually exclusive with ACC subtypes I or II.

### Safety and tolerability

Among all patients with ACC, five (26%) experienced a grade 3 to 5 AE regardless of treatment attribution. Table 2 describes the treatment-emergent AEs (TEAE) experienced by ≥ 15% of the ACC cohort or any grade 3 to 5 TEAEs. One (5%) patient had toxicity as the reason for study drug discontinuation, but no treatment-related deaths occurred. The most common TEAEs included nausea (14; 74%), vomiting (9; 47%), and fatigue (7; 37%). Elevated γ-glutamyl transferase was the only grade 3 treatment-related AE observed whereas no grade 4 treatment-related AEs occurred. Three (16%) patients interrupted drug dosing for grade 2 or less GI toxicity (diarrhea or nausea and vomiting).

**Table 2:** AEs

Event term	Number of patients (%) *N* = 19
Grade 3–5 AEs regardless of attribution	5 (26%)
AEs leading to treatment discontinuation	1 (5%)
TEAEs leading to death	0

TEAEs with a frequency of ≥15% or any grade 3–5	All	Grade 3	Grade 4	Grade 5
Nausea	14 (74%)	0	0	0
Vomiting	9 (47%)	0	0	0
Fatigue	7 (37%)	0	0	0
Elevated blood lactate dehydrogenase	5 (26%)	0	0	0
Diarrhea	5 (26%)	0	0	0
Constipation	5 (26%)	0	0	0
Dysgeusia	5 (26%)	0	0	0
Headache	5 (26%)	0	0	0
Proteinuria	4 (21%)	0	0	0
Decreased appetite	4 (21%)	0	0	0
Hyperuricemia	4 (21%)	0	0	0
Hypomagnesemia	4 (21%)	0	0	0
Hyponatremia	4 (21%)	0	0	0
Elevated aspartate aminotransferase	4 (21%)	0	0	0
Elevated lipase	4 (21%)	0	0	0
Elevated alanine aminotransferase	3 (16%)	0	0	0
Elevated blood alkaline phosphatase	3 (16%)	0	0	0
Dyspnea	3 (16%)	0	0	0
Cough	3 (16%)	0	0	0
Pruritus	3 (16%)	0	0	0
Hyperkalemia	3 (16%)	0	0	0
Anemia	3 (16%)	0	0	0
Elevated γ-glutamyl transferase	1 (5%)	1 (5%)	0	0
Aspiration pneumonia	1 (5%)	1 (5%)	0	0
Cancer-related pain	1 (5%)	1 (5%)	0	0
Cerebral empyema	1 (5%)	1 (5%)	0	0
Malignant pleural effusion	1 (5%)	1 (5%)	0	0

### Response and efficacy

**Figure 1:**
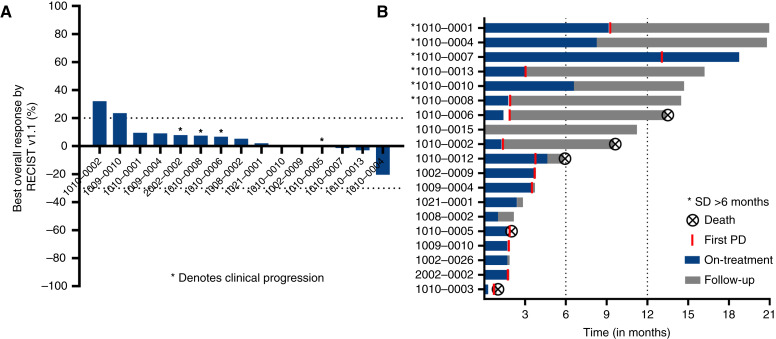
Response and efficacy. **A,** Waterfall plot depicting best overall response (RECIST v1.1) to KB-0742. Each column represents an individual patient. * denotes evidence of clinical progression of disease as best overall response. *N* = 15 as three patients were unevaluable and one had clinical progression prior to first restaging. Dotted lines mark the RECIST v1.1 thresholds for response and progression. **B,** Swimmer plot showing time on study treatments, point of progression, and follow-up period; each row represents an individual patient. An “X” within a circle represents a death event.

Among 16 evaluable patients, the best overall response rate was SD in nine (56%) patients with no observed complete or partial responses (Fig. 1A and B). A single patient had evidence of tumor regression (lasting 22.3 months; as second-line therapy for advanced disease). Seven (44%) experienced PD, including one unevaluable patient who discontinued therapy before first restaging for clinical PD. The median duration of disease stability was 3.96 months (95% CI, 1.56–9.48) with four of 16 (25%) experiencing SD for >6 months (range, 9.9–22.3 months) on treatment. Seven of the nine patients exhibiting SD had 0 to 1 prior lines of systemic therapy for advanced disease. One patient received treatment beyond progression for an additional 5 months. The primary reason for treatment discontinuation was PD (12; 63%), with one (5%) experiencing unacceptable toxicity (Supplementary Table S2). Patients received a median of two cycles of treatment (range, <1–10). Two patients withdrew consent from study participation and one was lost to follow-up.

### Survival outcomes

**Figure 2:**
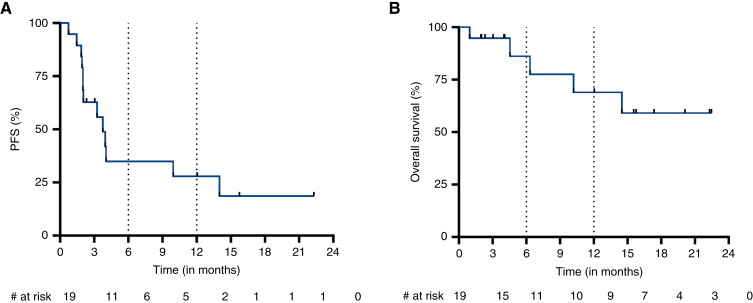
Survival outcomes. Kaplan–Meier survival curves plotting the probability of (**A**) PFS and (**B**) overall survival.

At a median follow-up of 6.3 months (range, 0.9–22.5), the median PFS was 3.8 months (95% CI, 2–14), with seven (44%) patients experiencing a progression event including one with clinical or radiologic evidence of PD (Fig. 2A). The 6-month and 12-month estimated PFS was 36.6% (95% CI, 14.2–59.8) and 29.3% (95% CI, 9.5–52.6), respectively. The median overall survival was not reached (range, 0.9–22.4 months) for the cohort, with five (26%) patients having died at last follow-up (Fig. 2B). The 6-month and 12-month estimated overall survival was 84.7% (95% CI, 49.7–96.1) and 75.3% (95% CI, 40.1–91.5), respectively.

### Molecular correlates

**Figure 3:**
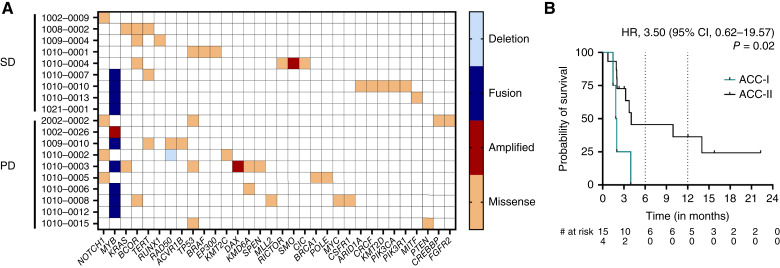
Molecular correlates.** A,** Tumor mutational landscape plot showing individual patient (row) and his/her next-generation sequencing data (columns) arranged by molecular event and group by best response to therapy. Somatic genomic event types are color coded. **B,** Kaplan–Meier survival curve plotting the probability of PFS separated by ACC-I vs. ACC-II phenotype.

All 19 patients had pretreatment tumor molecular profiling data available. Four (21%) had Notch pathway gain-of-function alterations, suggesting an ACC-I phenotype, whereas 10 (53%) had *MYB–NFIB* fusions or *MYB* amplification across both ACC phenotypes (Fig. 3A). No differences were observed in the tumor mutational profile between those patients who achieved a best response of SD versus progression, although three of four patients with ACC-I and an additional patient with ACC-II with a *MYC* gene amplification all experienced PD as best response. The median PFS was inferior among the patients with ACC-I (ACC-I: 1.9 vs. ACC-II: 4.0 months; HR, 3.50; 95% CI, 0.62–19.5; *P* = 0.02; Fig. 3B). Among patients with tumors harboring a *MYB* fusion, four (40%) exhibited a best response of SD.

## Discussion

Acknowledging the limited antitumor efficacy and toxicity profile of available therapies for recurrent or metastatic ACC, it is clear that investigating novel treatments remains a priority to address a significant unmet need. Given the importance of Notch signaling and its regulatory impact on TFs such as c-Myc, CDK9 represents an attractive therapeutic target given its role in transcriptional modulation (17). Although KB-0742 is a potent and selective inhibitor of CDK9, we did not observe responses among an advanced ACC population treated at the RP2D, suggestive of its limited clinical activity. Although pharmacodynamic data were limited for this expansion dataset, it is important to recognize the complexity of the *MYB* transcriptional regulatory program. One mechanism of potential resistance may have been high *MYB* expression counteracting the inhibitory effects of CDK9 inhibition (18). Furthermore, CDK9 kinase domain mutations can disrupt inhibitor binding, trigger compensatory pathways, and allow transcriptional recovery through epigenetic reprogramming (19) – all of which could have minimized antitumor efficacy.

Around half of evaluable patients with ACC exhibited SD with KB-0742. A closer look at these patients revealed that they often had the more indolent ACC-II phenotype, most had <2 prior lines of systemic therapy, few had experienced PD on their immediate prior line of treatment, and somatic alterations in chromatin remodeling genes were common (e.g., *BCOR*, *ARID1A*, *EP300*, and *KMT2D*). Furthermore, the median duration of SD was around 4 months although some experienced disease stability beyond 6 months. Recognizing the limitations of cross trial comparison, the median PFS was short at 3.8 months when reviewing other advanced ACC trial data (3). Recent trials exploring multitargeted VEGFR TKIs have yielded a median PFS closer to 7 to 9 months, but their tolerability can be limited by toxicity (7, 20, 21). It is important to note, however, that 25% of evaluable patients in the present study had an aggressive ACC-I phenotype which affected PFS, but mechanistically, CDK9 inhibition would have been predicted to benefit this subgroup the most.

Our data support that KB-0742 is a generally well-tolerated targeted therapy with most patients experiencing only mild GI toxicity, which certainly compares favorably when considering the side effect profile of VEGFR TKIs (6, 7, 9, 20, 21) and cytotoxic agents (4, 5). Similarly, γ-secretase inhibitors have modest activity in more aggressive cases of Notch activating ACC, but GI toxicity can be limiting (22). Understanding mechanisms of treatment resistance could lead to rational combinatorial strategies that incorporate CDK9 inhibitors or degraders given the tolerability and low discontinuation rate observed in the present study. CDK9 inhibition could lead to upregulation of antiapoptotic proteins like BCL-2, activation in the MAPK pathway, promotion of transcriptional signaling through compensatory activation of other CDKs, and epigenetic histone modifications. Any one of these proposed mechanisms of resistance could be targeted by combining CDK9 inhibitors with their respective small-molecule inhibitors (19, 23).

Limitations of the present study include the modest expansion cohort sample size of this rare tumor type although the clinical and molecular data available were detailed. Not requiring PD prior to enrollment also complicates the interpretation of disease stability and time to progression, but enrollment to the present study required progression or failure of the last line of therapy. PK, pharmacodynamic, and on-treatment molecular profiling data may be included in a future report summarizing the broader part 1 dose-escalation phase of the study. Given the lack of significant antitumor activity observed with KB-0742 in ACC, further clinical development in this disease setting seems unlikely unless rational combinatorial approaches are explored.

## Supplementary Material

Supplementary Table 1 Representativeness of Study Participants

Supplementary Table 2 Reasons for Treatment Discontinuation
