# Supplementary material for: Safety and Efficacy of a Selective Inhibitor of Cyclin-dependent Kinase 9 (KB-0742) in Patients with Recurrent or Metastatic Adenoid Cystic Carcinoma
Source: Cancer Res Commun. 2025 May 7;5(5):767–73. doi: 10.1158/2767-9764.CRC-25-0015 (PMC12056915; doi:10.1158/2767-9764.CRC-25-0015)
Supplement: Supplementary Table 1 — Representativeness of Study Participants [file crc-25-0015_supplementary_table_1_suppst1.docx]

**Supplemental Table 1. Representativeness of Study Participants**

| Cancer type(s)/subtype(s)/stage(s)/condition | Adenoid cystic carcinoma (ACC) |
| --- | --- |
| Considerations related to: | |
| Sex | ACC is more commonly diagnosed in women than in men, especially in certain locations like the breast. In contrast, ACC of the salivary glands might show a slightly higher prevalence in men, though gender differences are generally modest. In some studies, ACC has been reported to occur at a slightly younger age in women than in men, but the age range for diagnosis is typically broad, from young adults to older individuals. |
| Age | ACC can sometimes be diagnosed in younger individuals, including teenagers and people in their 20s or 30s, though this is less common. Most ACC cases occur in people between the ages of 40 and 60. This is the peak age range for diagnosis, especially for salivary gland ACC, which is the most common site for this cancer type. ACC can also occur in older individuals, including those in their 60s and 70s, though the incidence tends to decrease in this age group. |
| Race/ethnicity | - **Caucasians/White individuals**: ACC is most diagnosed in Caucasian populations, especially in Western countries such as the United States and Europe. This group is generally considered to have the highest incidence of ACC, particularly for salivary gland and breast ACC. - **Black/African American individuals**: There is less frequent reporting of ACC in Black populations, but the cancer does occur. In some studies, African Americans have been shown to have a slightly lower incidence of salivary gland ACC compared to White individuals. However, for ACC of the breast, the incidence in African American women appears to be lower than in Caucasian women as well. - **Asian populations**: ACC is less common in Asian populations compared to Caucasians, though it still occurs, particularly in the salivary glands. Some studies have shown that Asians might have a lower overall incidence of ACC, but in certain countries, such as Japan and China, salivary gland tumors—including ACC—are more frequently diagnosed compared to Western countries. However, the specific ethnic subgroup distribution within Asia varies. - **Hispanic/Latino populations**: Data on the incidence of ACC in Hispanic populations is sparse, but it is generally like that seen in Caucasian individuals. The rates of ACC in Hispanic individuals tend to align closely with overall trends in the general population. |
| Geography | Within individual countries, there may be regional differences in ACC incidence due to factors such as population density, access to healthcare, and environmental exposures. For example, in the U.S., urban areas may have higher diagnostic rates of ACC due to better access to specialized healthcare and cancer centers, while rural areas may report fewer cases, possibly due to lower awareness or access to diagnostic services.  The United States has a well-documented history of reporting ACC cases due to its extensive cancer registries and research. Incidence rates of ACC in the U.S. may be higher compared to some other countries, likely because of better diagnostic practices, as well as greater awareness and surveillance of rare cancers. |
| Other considerations | There are notable disparities in enrollment and participation in clinical trials for ACC. These disparities can impact the diversity of trial populations and the generalizability of findings. Here are some key factors contributing to these disparities:   - Socioeconomic Barriers: access to healthcare, cost to participate in trials, and health literacy. - Geographic Disparities: urban vs. rural, and international disparities. - Racial and Ethnic Disparities: underrepresentation of minorities, language, and communication barriers. - Awareness and Education: lack of awareness and physician referral patterns. - Trial Design and Inclusion Criteria: eligibility criteria strictness, and limited representation of rare cancer types. |
| Overall representativeness of this study | The age distribution of our study is like the average age distribution of ACC in the literature, median age of 66. Our study had limited race and ethnicity variation (100% White and a single Hispanic participant) despite our outreach efforts and engagement with underrepresented minorities to participate and minimizing study logistics, simplifying eligibility criteria, and providing translation of trial documents. The small sample size of the ACC population within the broader study must also be appreciated. |
