# Supplementary material for: Safety and Efficacy of a Selective Inhibitor of Cyclin-dependent Kinase 9 (KB-0742) in Patients with Recurrent or Metastatic Adenoid Cystic Carcinoma
Source: Cancer Res Commun. 2025 May 7;5(5):767–73. doi: 10.1158/2767-9764.CRC-25-0015 (PMC12056915; doi:10.1158/2767-9764.CRC-25-0015)
Supplement: Supplementary Table 2 — Reasons for Treatment Discontinuation [file crc-25-0015_supplementary_table_2_suppst2.docx]

**Supplemental Table 2. Reasons for Treatment Discontinuation**

| Reason | Number of patients (%)^A^ *N*=19 |
| --- | --- |
| Progression of disease | 12 (63) |
| Withdrawal of consent | 6 (32) |
| Toxicity | 1 (5) |
| Death^B^ | 0 |
| Subject non-compliance | 0 |

^A^ no subjects remain on-treatment at data cutoff; ^B^ no deaths were judged to be attributable to study treatment.
